# The Illusion of Moral Superiority

**DOI:** 10.1177/1948550616673878

**Published:** 2016-10-19

**Authors:** Ben M. Tappin, Ryan T. McKay

**Affiliations:** 1 ARC Centre of Excellence in Cognition and Its Disorders, Department of Psychology, Royal Holloway, University of London, Egham, Surrey, United Kingdom

**Keywords:** moral superiority, positive illusion, rationality, self-enhancement, social perception

## Abstract

Most people strongly believe they are just, virtuous, and moral; yet regard the average person as distinctly less so. This invites accusations of irrationality in moral judgment and perception—but direct evidence of irrationality is absent. Here, we quantify this irrationality and compare it against the irrationality in other domains of positive self-evaluation. Participants (*N* = 270) judged themselves and the average person on traits reflecting the core dimensions of social perception: morality, agency, and sociability. Adapting new methods, we reveal that virtually all individuals irrationally inflated their moral qualities, and the absolute and relative magnitude of this irrationality was greater than that in the other domains of positive self-evaluation. Inconsistent with prevailing theories of overly positive self-belief, irrational moral superiority was not associated with self-esteem. Taken together, these findings suggest that moral superiority is a uniquely strong and prevalent form of “positive illusion,” but the underlying function remains unknown.

Most people believe they are just, virtuous, and moral. These beliefs demand scientific attention for several reasons. For one, in contrast to other domains of positive self-belief, they likely contribute to the severity of human conflict. When opposing sides are convinced of their own righteousness, escalation of violence is more probable, and the odds of resolution are ominously low (Pinker, 2011; Skitka, Bauman, & Sargis, 2005). Moreover, self-righteousness is not confined to conflict situations; a substantial majority of individuals believe themselves to be morally superior to the average person. Compared to “above average” beliefs in other domains (e.g., see Alicke & Govorun, 2005), distinct lines of evidence suggest widespread moral superiority may be particularly irrational—yet, direct empirical support for this is absent. In the present study, we quantify this irrationality. We find that moral superiority represents a uniquely strong and prevalent instance of “positive illusion” (Taylor & Brown, 1988).

## Moral Superiority

In their seminal review, Taylor and Brown (1988) advanced the case for a triad of positive illusions—the first of which was overly positive self-evaluation. They regarded these phenomena as reflecting inaccuracies in social perception, persisting as a result of their beneficial effect upon human well-being. The common means of inferring the presence of positive illusions is to ask individuals how they compare with respect to the average person along some dimension. This method consistently reveals that an implausibly high number of people believe that they are above average—a phenomenon dubbed the “better-than-average effect” (Alicke & Govorun, 2005; or “self-enhancement,” Sedikides & Gregg, 2008). Although this phenomenon emerges across a range of characteristics, the magnitude of self-enhancement is strongest for moral qualities.

Across four studies, Alicke, Vredenburg, Hiatt, and Govorun (2001) reported evidence that desirable moral traits, such as honesty and trustworthiness, are associated with the largest difference between judgments of the self and the average person. A similar pattern has been found for undesirable traits—clearly moralized terms such as “liar” produce the strongest asymmetries in self-other judgment (Alicke, Klotz, Breitenbecher, Yurak, & Vredenburg, 1995). Multiple studies converge on the same conclusion: Magnitude of self-enhancement is stronger for moral characteristics—like honesty—than for other desirable but nonmoral characteristics, such as competence (Brown, 2012; Möller & Savyon, 2003), wisdom (Zell & Alicke, 2011), ambition (Alicke, Vredenburg, Hiatt, & Govorun, 2001), and intelligence (Van Lange & Sedikides, 1998). Moreover, whereas self-enhancement of various nonmoral traits may diminish with age, the self-other asymmetry for moral traits remains consistently large throughout the life span (Zell & Alicke, 2011). Such is the extent of this phenomenon that violent criminals consider themselves more moral than law-abiding citizens living in the community (Sedikides, Meek, Alicke, & Taylor, 2014).

## The Ubiquity of Virtue

To compound the paradox of widespread moral superiority, most individuals appear assured of the loftiness of their virtuous qualities (relative to their other qualities). Desirable moral traits are perceived to be highly descriptive of the self—more so than other desirable but nonmoral traits. For example, in a vast cross-national sample of 187,957 participants spanning 11 European countries, Gebauer, Wagner, Sedikides, and Neberich (2013) reported that, of two distinct trait dimensions, the one comprising desirable moral terms such as “faithful” and “honest” was judged as more self-descriptive than the one which included nonmoral terms such as “clever” and “wise.” Additional cross-cultural data converge on the same conclusion. In a similarly sized sample comprising participants from 54 countries and all 50 U.S. states, the moral characteristics of “honesty” and “fairness” were ranked consistently highly in individuals’ self-description (Park, Peterson, & Seligman, 2006). Wojciszke and Bialobrzeska (2014) compared two distinct trait dimensions, one including desirable moral characteristics—such as “fair,” honest, and “loyal”—and the other including desirable nonmoral characteristics such as “intelligent,” “knowledgeable,” and “logical.” Across six diverse cultures, they found that the traits in the former dimension were judged to be more descriptive of the self (also see Wojciszke, Baryla, Parzuchowski, Szymkow, & Abele, 2011). Indeed, numerous studies have shown that individuals believe they possess, on average, more honesty and trustworthiness than any other characteristic, including intelligence, modesty, friendliness, determination, and independence (e.g., Alicke et al., 2001; Brown, 2012; Sedikides, 1993). Finally, individuals anticipate that, whereas desirable nonmoral traits will come and go throughout the course of one’s life, they will always possess desirable moral traits (Ybarra, Park, Stanik, & Lee, 2012).

## The Paradox

Taken together, the preceding lines of evidence present a striking asymmetry. Most people consider themselves paragons of virtue; yet few individuals perceive this abundance of virtue in others. As a descriptive phenomenon, this pattern is perhaps unsurprising. Previous research indicates that self-enhancement emerges most strongly for traits that are both desirable and ambiguous (e.g., Dunning, Meyerowitz, & Holzberg, 1989)—a product of the increased degrees of freedom for self-favoring construal of the traits in question. That self-enhancement is strongest in the moral domain is directly consistent with this evidence. Morality traits are highly desirable (Van Lange & Sedikides, 1998) yet difficult to check against reality (Alicke & Govorun, 2005), and there is significant variability in the behaviors considered indicative of a “moral” person (Graham, Meindl, Beall, Johnson, & Zhang, 2016).

However, *normatively* speaking, moral superiority may reflect significant incoherence in social judgment and perception. To illustrate why, consider a typical individual, *Jane*, tasked with judging the morality of herself and the average person. The reviewed evidence suggests Jane construes her morality in very positive terms—in part by capitalizing on trait ambiguity. In contrast, her judgment of the average person is decidedly less positive. This suggests that Jane foregoes the corollary that high trait ambiguity permits a majority of others to be equally as moral as she, albeit in their own idiosyncratic ways (Dunning et al., 1989). Unfortunately, Jane’s double standard incurs a cost to her judgment accuracy. Self-judgments act as valid cues to what the average person is like—justified by the fact that most people are in the majority most of the time. Indeed, appropriately gauging the prototypicality of one’s own characteristics improves accuracy in judgments of ill-defined others (e.g., Hoch, 1987; Krueger & Chen, 2014); neglecting this prototypicality may thus amount to a failure of inductive reasoning (Krueger, Freestone, & MacInnis, 2013). Consequently, given that most people consider themselves highly moral, if Jane strongly self-enhances her morality—as the evidence indicates she will—this may also compromise the accuracy of her social perception.

## The Present Study

There is mounting support for the idea that moral superiority is an especially potent positive illusion. However, the term “illusion” specifically implies irrationality in belief—an accusation that lacks decisive evidence (Krueger & Wright, 2011). Prevailing measures of self-enhancement do not discriminate between the rational (i.e., defensible) and irrational (indefensible) components of self-enhancement (Heck & Krueger, 2015). To our knowledge, there have been no attempts to quantify and compare the irrationality in moral self-enhancement with that in other domains of self-enhancement. The present study addresses this lacuna. We adapt a novel method (Heck & Krueger, 2015) to isolate and quantify the irrational component of moral superiority and compare it against the irrationality in other domains of self-enhancement. We also examine whether the irrational component of moral superiority is associated with well-being, as the prevailing conception of positive illusions (Taylor & Brown, 1988), and previous research (e.g., Campbell, Rudich, & Sedikides, 2002), would predict.

## Method

### Participants

We sought to recruit 265 participants via Amazon’s Mechanical Turk (www.mturk.com) to achieve greater than 90% power to detect a small effect of *d* = 0.2 (at α = .05) in our primary analyses of variance and paired samples *t*-tests. We overrecruited by 15% to account for data exclusions, bringing our collected sample size to 308 participants (153 male; *M*_age_ = 37.81, *SD* = 11.77). Data from 20 participants were excluded from all analyses due to failing at least one attention check (8 participants) and/or providing incomplete responses (15 participants). A further 18 participants were excluded from the final regression analyses due to lack of variation in judgments of the self, the average person, and/or trait desirability—leaving a sample size of 270 for the primary analyses.

### Procedure and Materials

#### Procedure

After providing online consent, participants were presented with a list of 30 traits, comprising 10 trait terms each for the dimensions morality, agency, and sociability. They were asked to judge the extent to which each trait described (a) themselves, (b) the average person, and (c) the social desirability of each trait. Participants rated all 30 traits according to either (a), (b), or (c), before moving onto the next set of ratings, and the order of these three sets of judgments was counterbalanced across participants (any order effects were presumed to be trivial). The presentation order of the traits themselves was randomized across each rating set and participant. Rating judgments for the self and the average person were provided on a scale from 1 (*not at all*) to 7 (*very much so*). Social desirability judgments were also provided on a 7-point scale, ranging from −3 (*very undesirable*) to +3 (*very desirable*). Following the trait judgments, participants completed four other measures (counterbalanced, detailed below) and provided simple demographic information.

#### Traits

The core dimensions of social perception, *communion/warmth* and *agency/competence*, are associated with traits related to benevolence and ability, respectively (Fiske, Cuddy, & Glick, 2007). Recently, it has been empirically demonstrated that the communion/warmth dimension is comprised of distinct *morality* and *sociability* components—the former describing honesty, trustworthiness, and sincerity, the latter warmth, friendliness, and likeability (e.g., Goodwin, Piazza, & Rozin, 2014). Thus, drawing upon a comprehensive norming study of trait adjectives (Goodwin et al., 2014, Experiment 1), we selected five positive (desirable) and five negative (undesirable) traits for each of the three dimensions morality, agency, and sociability; providing a total of 30 traits for the present study. Traits were carefully chosen to minimize dimension overlap (see the Supplemental Online Material for details of the trait selection procedure).

#### Other measures

Self-esteem was measured using the 10-item Rosenberg Self-Esteem Scale (Rosenberg, 1965). Three additional measures were included but were not part of the primary analyses and are thus not reported further in the main text (see Supplemental Online Material for relevant analyses). These were the 16-item Narcissistic Personality Inventory (Ames, Rose, & Anderson, 2006), and the moral identity (Aquino & Reed, 2002), and need to belong (Leary, Kelly, Cottrell, & Schreindorfer, 2013) scales.

##### Projection-based index of self-enhancement

Prevailing measures of self-enhancement conflate defensible (or “rational”) self-enhancement with indefensible (or “irrational”) self-enhancement (Krueger & Wright, 2011). For instance, given that individuals have more information about themselves than about others, they will be relatively less certain about what the average person is like. As a consequence, judgments of the average person are likely to be less extreme than self-judgments; with the former tending toward the midpoint of the judgment scale (Moore & Small, 2007). The corollary is that observed self-other differences in trait judgment—ostensibly indicative of self-enhancement—may actually reflect rationally cautious judgments made under uncertainty. In order to estimate the irrational component of self-enhancement, it is therefore necessary to first account for this rational component. To this end, we adapted the Social Projection Index (SPI) of self-enhancement (Heck & Krueger, 2015).

To estimate what proportion of conventional self-enhancement may be considered rational, the SPI first asks how an individual might infer the characteristics of the average person. One strategy is to draw upon a relative abundance of self-knowledge. Indeed, in the absence of salient diagnostic information about others, one’s own characteristics act as cues to what others are like. Decades of research has shown that individuals readily project their own characteristics onto others and that this process—termed social projection—typically increases accuracy in judgments of what unknown others are like (for reviews, see Krueger, 2007; Robbins & Krueger, 2005). However, projection may be too weak or too strong—individuals may underperceive or overperceive the similarity between themselves and others, respectively. Somewhere in between is the optimal amount of projection, which tracks the *actual* similarity among people. While individuals are unlikely to perceive this similarity with complete precision, the researcher can. The similarity may be quantified as the correlation between individual self-judgments and the average of all self-judgments in the group (Hoch, 1987; Krueger et al., 2013). This correlation “coefficient of similarity” thus describes how typical of the average a particular individual is, and, importantly, a fully rational perceiver may weight their self-judgments by this coefficient to maximize accuracy in their judgments of what the average person is like (Hoch, 1987; Krueger & Chen, 2014). Computation of the coefficients of similarity, therefore, provides a rational benchmark against which to evaluate individuals’ observed self-enhancement (i.e., the difference between their self-judgments and their judgments of the average person).

To illustrate, consider the following example. An individual whose self-judgments are highly typical of the average should project more, as their self-judgments are highly diagnostic of what the average person is like. In contrast, an individual whose self-judgments are highly *a*typical of the average should project less, as their self-judgments are only weakly diagnostic of what the average person is like. In the former case, judgments of the average person are expected to be minimally regressive with respect to self-judgments—resulting in a smaller latitude for defensible (rational) self-enhancement. In contrast, in the latter case, judgments of the average person are expected to be relatively more regressive with respect to self-judgments—resulting in a larger latitude for defensible (rational) self-enhancement. Crucially, in either case, the SPI explicitly models the fact that informational uncertainty mandates that a proportion of conventional self-enhancement be considered rational—avoiding the pitfall of earlier measures.

Computationally speaking, the logic outlined above allows researchers to generate rational *predicted judgments of the average person*, by weighting individuals’ self-judgments by their respective coefficient of similarity. As Heck and Krueger (2015) point out however, this conceptualizes self-enhancement as diminishment of others. Alternatively, it is possible to reverse-predict what self-judgments *should* have been, if rationally projecting individuals derived their empirically observed judgments of the average person from their self-judgments. These reverse-predicted self-judgments may be labeled *inferred self-judgments*, and they yield a more conceptually appropriate interpretation of self-enhancement as positive self-inflation.

Determining the rational and irrational components of conventional self-enhancement thus requires self-judgments, judgments of the average person, and computed inferred self-judgments. Then, in a final step, the SPI exploits the empirical observation that most individuals possess a positive self-image; ascribing positive traits more readily to themselves than to others (and vice versa for negative traits). Accordingly, self-enhancement is modeled as the relationship between trait desirability and trait judgment,^1^ meaning that (a) conventional self-enhancement is given as the difference between how well trait desirability predicts self-judgments compared to how well it predicts judgments of the average person, (b) the rational component of self-enhancement is the difference between how well trait desirability predicts inferred self-judgments compared to how well it predicts judgments of the average person, and finally, (c) the irrational component of self-enhancement is the difference between how well trait desirability predicts inferred self-judgments compared to how well it predicts actual self-judgments.

## Results

### Descriptives

Table 1 displays the list of 30 traits, their mean self (S), average person (or “other,” O), and desirability (D) judgments, as well as the respective domain reliability coefficients. Table 2 displays the zero-order correlations among mean self, other, and desirability judgments for each trait domain.

**Table 1.** Mean Self, Other, and Desirability Trait Judgments and Domain Reliability Coefficients.

Trait	Self	Other	Desirability
Agency			
Hard working	5.71 (1.24)	4.44 (1.09)	6.54 (0.77)
Knowledgeable	5.66 (1.03)	4.27 (1.08)	6.37 (0.91)
Competent	5.89 (1.04)	4.49 (1.07)	6.48 (0.89)
Creative	4.87 (1.63)	3.94 (1.11)	5.90 (1.02)
Determined	5.66 (1.32)	4.54 (1.16)	6.18 (0.94)
Lazy	2.59 (1.54)	3.56 (1.29)	1.64 (1.04)
Undedicated	2.08 (1.36)	3.20 (1.21)	1.63 (0.91)
Unintelligent	1.63 (1.00)	3.34 (1.26)	1.56 (0.93)
Unmotivated	2.42 (1.50)	3.30 (1.22)	1.57 (0.93)
Illogical	2.02 (1.24)	3.56 (1.40)	1.72 (1.11)
*M*	3.85 (1.83)	3.87 (0.53)	3.96 (2.47)
Reliability (α)	.88	.93	.88
Sociability			
Sociable	4.31 (1.72)	4.99 (0.91)	6.25 (0.94)
Cooperative	5.50 (1.29)	4.64 (1.09)	6.41 (0.79)
Warm	5.13 (1.49)	4.48 (1.09)	6.41 (0.85)
Family orientated	4.98 (1.89)	4.83 (1.12)	5.87 (1.17)
Easy going	5.31 (1.45)	4.31 (1.02)	6.01 (1.02)
Cold	2.54 (1.57)	3.13 (1.15)	1.60 (1.02)
Disagreeable	2.38 (1.34)	3.32 (1.24)	1.49 (0.91)
Rude	2.14 (1.33)	3.34 (1.28)	1.29 (0.75)
Humorless	1.88 (1.24)	3.01 (1.12)	1.68 (1.02)
Uptight	2.47 (1.44)	3.47 (1.23)	1.83 (1.10)
*M*	3.66 (1.50)	3.95 (0.77)	3.88 (2.44)
Reliability (α)	.89	.88	.82
Morality			
Honest	5.93 (1.06)	4.44 (1.19)	6.55 (0.81)
Trustworthy	6.10 (0.98)	4.30 (1.26)	6.67 (0.78)
Fair	5.94 (0.99)	4.51 (1.13)	6.51 (0.85)
Respectful	5.88 (1.12)	4.55 (1.15)	6.52 (0.75)
Principled	5.63 (1.23)	4.26 (1.15)	6.16 (0.98)
Insincere	1.80 (1.02)	3.32 (1.31)	1.49 (0.90)
Prejudiced	2.12 (1.32)	3.78 (1.38)	1.51 (1.00)
Disloyal	1.65 (0.89)	3.06 (1.23)	1.31 (0.69)
Manipulative	2.10 (1.26)	3.39 (1.28)	1.60 (1.06)
Deceptive	2.07 (1.30)	3.34 (1.29)	1.44 (0.85)
*M*	3.92 (2.09)	3.89 (0.58)	3.98 (2.65)
Reliability (α)	.88	.93	.88
*M* (total)	3.81 (1.76)	3.90 (0.61)	3.94 (2.43)

*Note*. *N* = 288. For desirability judgments, the −3 to +3 scale was converted to 1–7. Standard deviations are given in parentheses.

**Table 2.** Zero-Order Correlations Among Mean Self, Other, and Desirability Judgments for Each Trait Domain.

Mean judgment	1	2	3	4	5	6	7	8	9
1. Agency, self	–								
2. Agency, other	.30	–							
3. Agency, desirability	.39	.19	–						
4. Sociability, self	.63	.47	.30	–					
5. Sociability, other	.24	.83	.15	.38	–				
6. Sociability, desirability	.36	.20	.66	.40	.21	–			
7. Morality, self	.65	.32	.38	.69	.27	.43	–		
8. Morality, other	.23	.87	.16	.43	.89	.20	.30	–	
9. Morality, desirability	.39	.20	.74	.35	.16	.74	.46	.20	–

*Note*. *N* = 288. For meaningful interpretation, the coefficients are based upon means calculated after reverse-coding negative traits.

All *p*s < .05.

### Rational and Irrational Self-Enhancement

To compute the rational and irrational components of self-enhancement, we first calculated the similarity between individual self-judgments and the average of all self-judgments in the group (i.e., the coefficients of similarity for each participant). Thus, for each participant, we regressed the average self-judgments made by all participants for the traits in a given dimension onto the self-judgments of the focal participant for the traits in that dimension. Estimating unique coefficients for each dimension acknowledges that individuals may be more similar on some dimensions compared to others. We thus obtained three coefficients of similarity and their corresponding intercepts for each participant. As outlined above, the SPI posits that these coefficients may be used to weight S judgments to generate rational predicted O judgments, P. P is thus given as:

P=coefficient of similarity∗S judgment+intercept.

However, following Heck and Krueger (2015), we instead computed inferred self-judgments, I, by rewriting the regression equation:

$$\text{I}=\frac{\text{O}\;\text{judgment}}{\text{coefficient}\;\text{of}\;\text{similarity}}+\text{intercept.}$$

We computed I judgments for each trait over all participants, using the mean^2^ coefficient of similarity and intercept corresponding to the traits’ respective dimension:

$$\text{I}=\frac{\text{O}}{.85}+.61\;[\text{Morality}\;\text{traits}].$$

$$\text{I}=\frac{\text{O}}{.73}+1.04\;[\text{Agency}\;\text{traits}].$$

$$\text{I}=\frac{\text{O}}{.52}+1.77\;[\text{Sociability}\;\text{traits}].$$

Thus, at this stage, each participant had four sets of judgments for the 30 traits: Their empirically observed S, O, and D judgments and the new I judgments computed according to the method outline above. For each dimension, we then regressed S, O, and I on D judgments over each participant. We also regressed O on S judgments to cross-check the assumption of social projection (a positive association constitutes evidence for social projection; Krueger, 2007). Table 3 displays the relevant means. We first draw attention to the evidence of social projection; across all trait dimensions, S judgments positively predicted O judgments (mean *b*_SO_ = .23–.30). We then examined whether social projection increased judgment accuracy. For each dimension, we computed an accuracy index by correlating other judgments with average self-judgments over all participants and traits corresponding to that dimension. Correlating this index with magnitude of social projection revealed the expected pattern. Accuracy of other judgments was positively associated with social projection across all trait dimensions, *r*(268) = .63–.85, all *p*s < .001. As projection increased, accuracy of other judgments improved. This is consistent with previous research (Hoch, 1987; Krueger & Chen, 2014) and confirms the validity of the methodological approach taken in the present study.

**Table 3.** Mean Slopes and Intercepts From Primary Regression Analyses.

	Unstandardized				Standardized	
Regressions by trait domain	Slope (*b*)		Intercept		β	95% CI [LL, UL]
	*M*	*SE*	*M*	*SE*	*M*	
Agency						
*R*_SD_	.70	.02	1.06	.09	.80	[0.76, 0.83]
*R*_OD_	.21	.02	3.04	.10	.38	[0.30, 0.45]
*R*_SO_	.23	.02	3.01	.10	.36	[0.29, 0.44]
*R*_ID_	.28	.03	5.17	.14	.38	[0.30, 0.45]
Sociability						
*R*_SD_	.56	.02	1.48	.09	.67	[0.61, 0.72]
*R*_OD_	.30	.02	2.78	.09	.55	[0.49, 0.61]
*R*_SO_	.30	.02	2.81	.10	.45	[0.39, 0.51]
*R*_ID_	.57	.04	7.13	.17	.55	[0.49, 0.61]
Morality						
*R*_SD_	.76	.02	0.92	.07	.88	[0.85, 0.90]
*R*_OD_	.21	.02	3.02	.09	.41	[0.33, 0.48]
*R*_SO_	.25	.02	2.90	.10	.41	[0.34, 0.48]
*R*_ID_	.25	.03	4.17	.11	.41	[0.33, 0.48]

*Note*. *N* = 270. Unstandardized slopes (*b*) are used in analyses. *R* = regression; S = self; O = other; D = desirability; I = inferred self; *SE* = standard error; CI = confidence interval; LL = lower limit; UL = upper limit.

Next, we note the expected observation of conventional self-enhancement. Across all dimensions, trait desirability predicted self-judgments (mean *b*_SD_) better than it predicted other judgments (mean *b*_OD_); for morality (.76 vs. .21), *t*(269) = 22.08, *p* < .001, *d* = 1.34 95% confidence interval (CI) [1.18, 1.51], agency (.70 vs. .21), *t*(269) = 18.75, *p* < .001, *d* = 1.14 [0.99, 1.29], and sociability (.56 vs. .30), *t*(269) = 10.21, *p* < .001, *d* = 0.62 [0.49, 0.75]. To compare across dimensions, we computed a difference measure of conventional self-enhancement as *b*_SD_ − *b*_OD_ and conducted a repeated measures analysis of variance with dimension as the single factor, *F*(2, 538) = 71.70, *p* < .001, η_p_^2^ = .21. Conventional self-enhancement was greater for morality (.54) than agency (.49), *t*(269) = 2.01, *p* = .045, *d* = 0.12 [0.00, 0.24], and sociability (.26), *t*(269) = 11.04, *p* < .001, *d* = 0.67 [0.54, 0.80]. Agency was also greater than sociability, *t*(269) = 8.42, *p* < .001, *d* = 0.51 [0.39, 0.64].

What proportion of conventional self-enhancement is accounted for by rational and irrational components? To determine the rational component, we examined how well trait desirability predicted inferred-self judgments (mean *b*_ID_) compared to other judgments (mean *b*_OD_). Sociability had the largest magnitude of rationally defensible self-enhancement (.57 vs. .30), *t*(269) = 14.33, *p* < .001, *d* = 0.87 [0.73, 1.01]. The magnitude for agency was substantially smaller (.28 vs. 21), but still nontrivial, *t*(269) = 3.19, *p* = .002, *d* = 0.19 [0.07, 0.31]. In contrast, the rational component of self-enhancement in the moral domain was trivial in size (.25 vs. .21), *t*(269) = 1.72, *p* = .087, *d* = 0.10 [−0.02, 0.22].

**Figure 1.**
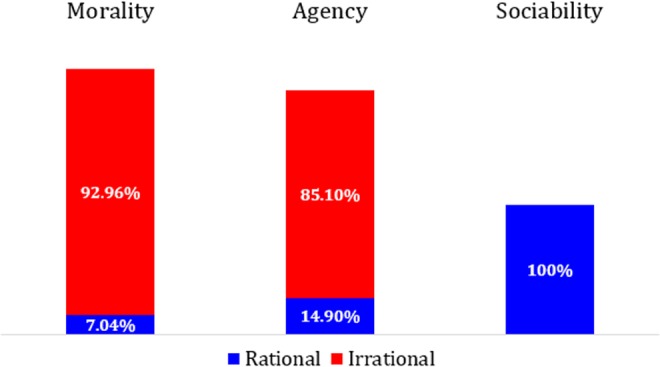
Percentage of conventional self-enhancement (*b*_SD_ − *b*_OD_) magnitude accounted for by rational (*b*_ID_ − *b*_OD_) and irrational (*b*_SD_ − *b*_ID_) components as a function of trait dimension. *N* = 270.

This indicates that irrational self-enhancement is strongest in the moral domain. Indeed, this was the case. Examining how well trait desirability predicted actual self-judgments (mean *b*_SD_) compared to inferred self-judgments (mean *b*_ID_) revealed the largest discrepancy for morality (.76 vs. .25), *t*(269) = 18.17, *p* < .001, *d* = 1.11 [0.95, 1.26]. Although smaller, agency comprised a substantial magnitude of irrational self-enhancement (.70 vs. .28), *t*(269) = 12.97, *p* < .001, *d* = 0.79 [0.65, 0.93]. In stark contrast, there was no evidence for irrationality in self-enhancement of sociability traits—desirability predicted self and inferred self-judgments equally well (.56 vs. .57), *t*(269) = −0.32, *p* = .750, *d* = −0.02 [−0.14, 0.10]. In other words, the average magnitude of conventional self-enhancement along the dimension of sociability was fully accounted for by rational projection-based other judgment. As before, we compared irrationality across dimensions by computing a difference measure, *b*_SD_ − *b*_ID_, and conducting a repeated measures analysis of variance with dimension as the single factor, *F*(2, 538) = 187.19, *p* < .001, η_p_^2^ = .41. Morality (.50) comprised the greatest magnitude of irrational self-enhancement, compared with agency (.42), *t*(269) = 3.33, *p* = .001, *d* = 0.20 [0.08, 0.32], and sociability (−.01), *t*(269) = 18.04, *p* < .001, *d* = 1.10 [0.95, 1.25]. Agency was also greater than sociability, *t*(269) = 13.25, *p* < .001, *d* = 0.81 [0.67, 0.94]. The complete pattern is displayed in Figure 1 as the percentage of conventional self-enhancement magnitude accounted for by rational and irrational components.

Corroborating the analysis of magnitude, the data also show that more individuals irrationally self-enhanced (*b*_SD_ > *b*_ID_) for moral traits (243, 90% of sample), compared with agency traits (218, 81%), χ^2^(270) = 14.05, *p* < .001, and sociability traits (134, 50%), χ^2^(270) = 103.22, *p* < .001. Individuals were also more likely to irrationally self-enhance for agency traits than for sociability traits, χ^2^(270) = 73.29, *p* < .001.

### Ubiquity of Virtue

What accounts for the strength and prevalence of irrationality in moral self-enhancement? Consistent with the *ubiquity of virtue*, prototypicality of individual self-judgments was highest in the moral domain—self-judgments tracked average self-judgments better for morality traits (mean *b* = .85) than agency traits (.73), *t*(269) = 5.96, *p* < .001, *d* = 0.36 [0.24, 0.49], and sociability traits (.52), *t*(269) = 12.94, *p* < .001, *d* = 0.79 [0.65, 0.92]. In other words, as expected, the strongest consensus in self-judgment emerged in the moral domain. Importantly however, strength of projection to others was not adequately adjusted to reflect this consensus. Projection from self to other was no stronger in the moral domain (mean *b*_SO_ = .25) than in the agency domain (.23), *t*(269) = 1.51, *p* = .133, *d* = 0.09 [−0.03, 0.21], and was in fact *weaker* than projection in the domain of sociability (.30), *t*(269) = 3.07, *p* = .002, *d* = 0.19 [0.07, 0.31]. Thus, trait desirability predicted moral self-judgments to a much greater extent than it predicted moral other judgments, and, taken in conjunction with the ubiquity of virtue, this discrepancy is classified as largely irrational.

### Inaccuracy and Well-Being

The prevailing conception of positive illusions (Taylor & Brown, 1988) encompasses two central claims about strongly positive self-evaluations. The first is that they reflect an inaccurate perception of reality (i), and the second is that this inaccuracy contributes to well-being (ii).

To examine (i), for each dimension we correlated the previously computed accuracy index with magnitude of irrational self-enhancement in that dimension. As expected, accuracy was negatively associated with irrational self-enhancement; for morality, *r*(268) = −.73, *p* < .001, agency, *r*(268) = −.71, *p* < .001, and sociability *r*(268) = −.65, *p* < .001. The aforementioned accuracy index denotes *discrimination* accuracy only; thus we also examined *bias*—that is, absolute discrepancies in judgment (Epley & Dunning, 2006). For each dimension, we computed a discrepancy index of accuracy as the average difference between other judgments and the average of all self-judgments over traits and participants. We entered these scores into a repeated measures analysis of variance with dimension as the single factor, *F*(2, 538) = 122.67, *p* < .001, η_p_^2^ = .31. Judgments of others’ morality (1.56) were the most discrepant compared with agency (1.40), *t*(269) = 5.55, *p* < .001, *d* = 0.34 [0.22, 0.46] and sociability (1.10), *t*(269) = 15.11, *p* < .001, *d* = 0.92 [0.78, 1.06] judgments. The most inaccurate judgments of others occurred in the domain with the strongest irrationality in self-enhancement. Thus, both accuracy analyses support claim (i).

Moving onto (ii), we conducted partial correlations between irrational self-enhancement and self-esteem—controlling for the confounding influence of the corresponding rational, and the other dimensions’ rational and irrational, components of self-enhancement. Magnitude of irrational self-enhancement in the moral domain was *not* associated with self-esteem, *r*(263) = −.02, *p* = .701, whereas irrational self-enhancement in the agency and sociability domains was positively correlated with self-esteem, *r*(263) = .30, *p* < .001 and *r*(263) = .25, *p* < .001, respectively.

## Discussion

The present study revealed two key findings. The first was that moral superiority comprised a substantial irrational component; the absolute and relative magnitude of which was greater than that observed in other domains of self-enhancement. Indeed, virtually all individuals irrationally inflated their moral qualities. The second key finding was that, unlike the other domains of self-enhancement, irrational moral superiority was not associated with self-esteem. Taken together, these results suggest a uniquely strong and prevalent illusion of moral superiority and raise intriguing questions about the function of this phenomenon.

The irrationality of moral superiority was borne out of the ubiquity of virtue—almost everyone reported a strong positive moral self-image—and individuals’ ignorance of this ubiquity when making judgments of the average person. Indeed, neglecting the prototypicality—and thus cue validity—of one’s own self-judgments may signal an error in inductive reasoning (Krueger et al., 2013). Of course, self-judgments themselves may not accurately reflect genuine moral character—for example, compared to behavior (Back & Vazire, 2012). However, given the substantial degrees of freedom in what constitutes moral behavior (Alicke & Govorun, 2005; Graham et al., 2016), it seems probable that claims of positive moral character are equally legitimate (or illegitimate) for a large majority of people. In most cases, it would be difficult to make the argument that one moral self-image is more genuine than another. A fallacy thus arises when individuals do not apply to others the same degrees of freedom they invoke in their moral evaluation of themselves (Dunning et al., 1989). Insofar as this fallacy compromises the accuracy of social judgment and perception, it may be deemed erroneous (Heck & Krueger, 2015).

Despite finding strong support for the illusory nature of moral superiority, we found that the irrational component of moral self-enhancement was not correlated with self-esteem. This is inconsistent with the prevailing conception of positive illusions (Taylor & Brown, 1988) and is especially pronounced given that self-esteem was positively associated with magnitude of irrational superiority in both the agency and sociability domains. Furthermore, our result is at odds with previous evidence that high self-esteem individuals possess a stronger belief in moral superiority (Campbell et al., 2002). However, the latter inconsistency may be accounted for by measurement differences. Campbell and colleagues assessed superiority using a “comparative” measure, that is, they directly asked individuals how much better than average they are. These measures correlate most strongly with self-judgments, and only weakly with judgments of others (Klar & Giladi, 1999; Krueger & Wright, 2011)—the corollary being that the measure used by Campbell and colleagues may have assessed (absolute) moral self-image rather than (relative) moral superiority. Support for this proposition is recovered from our own data; self-esteem *did* positively correlate with moral self-image (morality *b*_SD_), *r*(268) = .34, *p* < .001.

As an indicator of well-being, self-reported self-esteem is far from exhaustive; it is necessary to measure well-being by more objective means to decisively test the theory of positive illusions (Heck & Krueger, 2015). Nevertheless, the lack of a relationship between self-esteem and the irrational component of moral superiority invites speculation as to why this illusion is so pervasive (cf. Taylor & Brown, 1988). Although a full discussion is beyond the scope of this article, we note that, from other perspectives, moral superiority may not be considered irrational at all (cf. Boudry, Vlerick, & McKay, 2015). For example, error management theorists (e.g., Haselton & Buss, 2000) might view underestimating the morality of others as quite rational. Mistaking another person as trustworthy, when in fact they are not, may be associated with greater fitness costs than the reverse error. Under such conditions, individuals may tolerate decreased judgment accuracy for gains made elsewhere (Fetchenhauer & Dunning, 2006; but see McKay & Efferson, 2010). On this account, moral superiority may persist, in part, as a function of the adaptive value of presuming modest morality in unknown others.

The findings of the present study are limited in that they do not reveal the behavioral consequences of an illusion of moral superiority. While we advance the case that moral superiority is dubious partly because “morality” may be defined by many different behaviors (Alicke & Govorun, 2005; Graham et al., 2016), it would be practically useful to know whether the illusion of moral superiority predicts certain types of moral behavior—for example, dishonesty for monetary gain. On the basis of existing research there is scope for competing predictions. Given the evidence that affirmation of moral image “licenses” subsequent immoral behavior (Blanken, van de Ven, & Zeelenberg, 2015), feeling morally superior may promote greater dishonesty. Alternatively, to the extent that people value belief-behavior consistency (Festinger, 1962), moral superiority may be associated with a greater likelihood of honest behavior. We defer to future research to test these hypotheses.

The belief that one is morally superior to the average person appears robust and widespread. Our examination of this belief revealed substantial irrationality beyond that observed in other domains of positive self-evaluation. On this basis, moral superiority represents a uniquely strong and prevalent form of positive illusion.

## Supplementary Material

Supplementary material

## Notes

1. This index of self-enhancement is psychometrically equivalent to a conventional difference-score index (see Heck & Krueger, 2015).

2. While a more fine-grained approach is to generate inferred self-judgments using the participants’ idiosyncratic coefficients of similarity—rather than the mean—these different approaches yield equivalent results overall (see, e.g., Heck & Krueger, 2015).
