# Supplementary material for: The Illusion of Moral Superiority
Source: Soc Psychol Personal Sci. 2016 Oct 19;8(6):623–31. doi: 10.1177/1948550616673878 (PMC5641986; doi:10.1177/1948550616673878)
Supplement: Supplementary material [file SPPS673878_suppl_mat.pdf]

Supplemental Material: The Illusion of Moral Superiority

Method

Procedure & Materials.

Traits. Goodwin et al. (2014, Experiment 1) asked 1,048 respondents how useful trait adjectives (170 total) were in providing information about higher-level person characteristics—such as "ability", "morality", and "character". Participants also rated the valence of the traits. For the present study, we selected traits from this dataset according to the following procedure. First, we averaged across relevant characteristics to obtain composite mean ratings for *morality* ("morality/immorality", "character"), *agency* ("ability", "agency"), and *sociability* ("warmth", "communion") dimensions. Next, for each dimension we identified 10 traits with high composite mean ratings for that dimension; 5 positively valenced (i.e., desirable) and 5 negatively valenced (undesirable). We selected both desirable and undesirable traits for the present study because the regression-based index of self-enhancement that we adapted (outlined in the main text) models self-enhancement as the relationship between trait desirability and trait judgment. Thus, including desirable and undesirable traits generates the required variability in trait desirability judgments. We avoided traits that had a strong overlap across dimensions—for example, "compassionate" garnered an equally high composite rating for *morality* (7.55) and *sociability* (7.71), and was therefore not selected for either dimension. We also avoided direct antonyms (e.g., inclusion of both "honest" and "dishonest") and synonyms (e.g., "unintelligent", "stupid"). Two traits, "manipulative" and "deceptive", were not included in Goodwin et al. (2014), but were added to our final list of morality traits.

Results

Self-centrality breeds self-enhancement. Gebauer and colleagues (2013) reported evidence that domain self-centrality predicts stronger magnitude of conventional self-enhancement in that domain. Thus, we explored whether the self-centrality of a given trait domain would predict greater magnitude of *irrational* self-enhancement in that domain. Based upon the rationale behind the constructs, we included the Narcissistic Personality Inventory (NPI-16, Ames et al., 2006), Need-to-belong (NTB, Leary et al., 2013) and Moral Identity (Aquino & Reed, 2002) scales as measures of the self-centrality of agency, sociability, and morality, respectively. All scale items loaded onto their predicted number of factors (all factor loadings ≥.36.), and the scales demonstrated acceptable reliabilities (α's = .86-.92). We thus computed sum scores for each scale following the authors' respective instructions.

We then conducted partial correlations[1] between construct scores and their respective irrational self-enhancement domain. Neither the *internalization* subscale nor overall moral identity scores were related to magnitude of irrational self-enhancement in the moral domain, r (263) = .08, p=.174, and r (263) = -.08, p=.208, respectively. Interestingly, the *symbolization* subscale demonstrated a small negative association with irrational moral superiority; r (263) = -.17, p=.005. We also found that scores on the NPI positively correlated with irrational self-enhancement in the domain of agency; r (263) = .35, p<.001. This is consistent with previous research which reported that narcissists most strongly self-enhance their agency characteristics (e.g., Campbell et al., 2002). Finally, irrational self-enhancement of sociability traits did not relate to NTB

[1] Controlling for the corresponding rational, and other dimensions' rational and irrational, components of self-enhancement.

scores, r (263) = .03, p=.645. Thus, we found limited evidence that self-centrality—as construed by the included construct scales—breeds irrational self-enhancement.
